# Sleep Supports Memory of Odors in Adults but Not in Children

**DOI:** 10.1371/journal.pone.0139069

**Published:** 2015-09-25

**Authors:** Alexander Prehn-Kristensen, Kristin Lotzkat, Eva Bauhofer, Christian D. Wiesner, Lioba Baving

**Affiliations:** 1 Department of Child and Adolescent Psychiatry and Psychotherapy, Center for Integrative Psychiatry, School of Medicine, Christian-Albrechts-University, Kiel, Germany; 2 Department of Psychology, Christian-Albrechts-University, Kiel, Germany; Université Lyon, FRANCE

## Abstract

Sleep supports the consolidation of declarative memory in children and adults. However, it is unclear whether sleep improves odor memory in children as well as adults. Thirty healthy children (mean age of 10.6, ranging from 8–12 yrs.) and 30 healthy adults (mean age of 25.4, ranging from 20–30 yrs.) participated in an incidental odor recognition paradigm. While learning of 10 target odorants took place in the evening and retrieval (10 target and 10 distractor odorants) the next morning in the sleep groups (adults: n = 15, children: n = 15), the time schedule was vice versa in the wake groups (n = 15 each). During encoding, adults rated odors as being more familiar. After the retention interval, adult participants of the sleep group recognized odors better than adults in the wake group. While children in the wake group showed memory performance comparable to the adult wake group, the children sleep group performed worse than adult and children wake groups. Correlations between memory performance and familiarity ratings during encoding indicate that pre-experiences might be critical in determining whether sleep improves or worsens memory consolidation.

## Introduction

The olfactory system is characterized by remarkable plasticity, and odors have a strong ability to spontaneously evoke emotional and autobiographical memories [1,2,3]. Some associations between odors and autobiographical episodes endure lifelong even without rehearsing [4]. Responses to the majority of odors, however, are not inherent but experience-dependent, and, during infancy, most odors are unfamiliar and meaningless until they become associated with personal experiences [5].

There is convincing evidence that sleep supports the consolidation of declarative memories (i.e. episodic or semantic memory) in adults as well as in children [6,7]. Newly encoded information is stored temporarily in the hippocampus; during post-learning slow wave sleep (SWS) hippocampal representations become integrated into pre-existing neocortical memory systems, leading to better memory performance for personal episodes, word-pair or object-location association tasks after sleep [8,9,10].

Despite the close relationship between odor and associative memory, only little is known about possible interaction between sleep and odor memory. In rats it has been shown that sleep enhances the associations between odors and threatening stimuli in a fear-conditioning paradigm [11,12]. In most human studies, odors were used as contextual cues that can support the consolidation of declarative memories during sleep. Object locations were learned while a certain odor was presented; better memory performance after sleep was observed when the same odor was presented during post-learning sleep [13,14]. So far, only one human study [15] investigated the role of sleep in the consolidation of odor memory. It was found that odors were recognized better after 3-hours of SWS-rich sleep than after wakefulness. In that study, however, memory instructions were given explicitly: during encoding participants were aware of the fact that they were to remember as many odors as possible for later on. Successful intentional odor learning, in turn, is closely related to the strategy of verbally labeling odors [16,17]. Therefore, it cannot be ruled out that in the study by Gais and colleagues [15] sleep fostered the memory of odor sensation itself or rather the recognition of declarative odor labels cued by odors.

Sleep changes over time. The most dramatic changes in sleep patterns can be found during the transition from childhood to adulthood. Here, a delay of the sleep-wake cycle, a decrease of total sleep time accompanied by a reduction of SWS, and dynamic changes in sleep spindle activity take place [18,19,20,21,22]. Since SWS is causally linked to memory consolidation, school children can outperform adults with respect to declarative memory performance [23]. In contrast, motor memory consolidation, which has been shown to be supported by sleep in adults [24,25] is not fostered by sleep in children [26,27,28]. Memory consolidation during sleep depends on the presence of pre-existing memory representations [29,30], and it is therefore assumed that sleep does not support the consolidation of motor memory in children due to fewer motor experiences [6].

The aim of this study was to examine whether sleep fosters basal odor memory in adults as well as in children. Since intentional odor learning can be confounded by declarative memory aspects (e.g. memorizing odor labels rather than the odor perception itself), an incidental odor learning task with unannounced subsequent recognition was chosen.

## Materials and Methods

### Participants

All adult participants gave written informed consent, and in case of children, written informed consent was obtained by children and their caretakers. All participants were reimbursed with a voucher for their participation. The study was approved by the ethics committee of the medical faculty of the University of Kiel, Germany.

Thirty boys (mean age of 10.6, ranging from 8–12 yrs.) and 30 male college students (mean age of 25.4, ranging from 20–30 yrs.) participated in this study (for participants´ characteristics see also Table 1). All participants were non-smokers (self-report), free of chronic or acute physical illnesses (self-report), psychiatric diseases (children: Child Behavior Checklist, CBCL [31], cut off: t>67; adults: Symptom Check List 90 revised, SCL-90-R [32], cut off: Global severity index t>63), alcohol or drug abuse (self-report), shift work, or sleep disorders (children: Children´s Sleep Habits Questionnaire, CSHQ [33], cut off: sum score>44; Pittsburg Sleep Quality Index, PSQI [34], cut-off>5). Sleep questionnaires revealed that the usual time to wake up in adults was on average about 70 minutes later than in children (adults: sleep group: M = 7:40 a.m., wake group: M = 7:49 a.m.; sleep vs. wake: p = .6; children: sleep group: 6:31 a.m., wake group: 6:34 a.m.; sleep vs. wake: p = .8). None of them displayed impaired sense of smell which had been determined by a three-alternative, forced-choice odor discrimination test. In each of three trials, participants were to detect a target out of two distractor odorants; alpha-error: p = .027). Pubertal development in children was assessed by the German version of the Pubertal Development Scale (PDS) [35]. According to the PDS parental ratings, 26 children were prepubertal, 3 were early pubertal, and 1 mid-pubertal. Children and adults were assigned randomly either to a wake group or a sleep group (n = 15 per group). Neither the sleep/wake groups of adults nor the sleep/wake groups of children differed with respect to age (children: p = .694; adults: p = .779), psychiatric symptoms (children´s CBCL sum scores: p>.5; adults´ SCL-90-R sum scores: p>.7), or sleep behavior (children´s CSHQ sum score: p = .694; adults´ PSQI sum score p = .668). In addition, the children’s groups did not differ in puberty stage (p = .539). For all group comparisons, t-tests were used except for the PDS sore, where the Mann-U-test was applied.

**Table 1:** Participant characteristics, odor memory performance.

	Adult			Children		
	Sleep Group	Wake Group	Sleep vs. Wake	Sleep Group	Wake Group	Sleep vs. Wake
	M (SEM)	M (SEM)	p-value	M (SEM)	M (SEM)	p-value
Age (yrs.)	25.6 (0.68)	25.3 (0.78)	.696	10.7 (0.4)	10.5 (0.32)	.779
Psychiatric symptoms (t-scores) Adults: SCL, Children: CBCL	45.1 (1.6)	44.9 (1.4)	.951	44.9 (2.2)	43.2 (1.9)	.562
Sleep behavior Adults: PSQI, Children: CSHQ	3.1 (0.39)	2.9 (0.25)	.668	38.5 (2.7)	38.9 (2.8)	.694
TST actigraphy (h)	7.8 (0.27)	-	-	8.8 (0.27)	-	-
Recognition Accuracy (d´)	1.62 (0.14)	1.15 (0.17)	.038	0.8 (0.16)	1.38 (0.12)	.008

Note: SCL, Symptom Check List: Global Severity Index; CBCL, Child Behavior Check List: Sum Score; PSQI, Pittsburg Sleep Quality Index: Sum Score; CSHQ, Children’s Sleep Habit Questionnaire: Sum Score; TST, Total Sleep Time.

### Stimulus material

Twenty odorants (scent-impregnated patches) of the University of Pennsylvania Smell Identification Test (UPSIT, Sensonics, Inc., NJ, USA) were used for the odor recognition paradigm: Ten target odorants from four odor categories (fruit: banana, apple, orange; food: pizza, fruit punch; non-food: gasoline, smoke; spice/plant: pine, lilac, cinnamon) and ten distracting odorants from the same categories were chosen (fruit: cherry, coconut, pineapple; food: onion, bubble gum; non-food: paint thinner, leather; spice/plant: cedar, rose, menthol).

### Procedure

All participants were told that they were taking part in a study investigating the circadian effects on odor perception. During encoding, participants were seated in an air-conditioned lab and asked to rate each of the ten target odorants with respect to their intensity, pleasantness, unpleasantness, and familiarity on 9-point analogue scales (from 0 = “not at all” to 8 = “maximum”). Participants were blinded by a sleeping mask. No memory instructions were given and participants were instructed to describe odors solely along the rating scales without any further personal reports (e.g. odor names, individual associations). Twelve hours later, participants returned to the lab, and the retrieval session took place, in which target and distractor odorants were presented in pseudorandomized order under the same environmental conditions as during encoding. After smelling each odorant, participants were asked to indicate whether or not they recognize the current odorant from the encoding session by verbally indicating either “old” or “new”. Hereafter, ratings of odorants´ intensity, valence, and familiarity were requested. At the end of the retrieval session, participants were asked if they suspected being part of a memory study. If the answer was “yes”, participants were asked to rate their confidence on an analogue scale (from 0 = “not at all” to 100 = “absolutely sure”).

In the sleep group the encoding session took place in the evening (in children: 8 p.m., in adults: 9 p.m.) and the retrieval session the next morning (in children: 8 a.m., in adults: 9 a.m.) after a night of sleep; in the wake group the time schedule was vice versa, with the exception that no sleep was allowed within the 12-hour retention interval. Participants were asked to refrain from eating or drinking flavored drinks at least 30 min prior to each experimental session. In the beginning of each of the experimental sessions, participants´ current sense of smell was assessed by using the abovementioned three-alternative, forced-choice odor discrimination test. Then, participants were asked to rate their current mood (ranging from 1 = “feeling down” to 6 = “feeling happy”), tiredness (ranging from 1 = “feeling tired” to 6 = “feeling refreshed”), arousal (ranging from 1 = “feeling tense” to 6 = “feeling relaxed”) and the quality/quantity of the last sleep episode by using a standard evening and morning protocol [36]. Participants of the sleep group were asked to maintain their usual bedtimes. All participants were told to wear an actigraph (SOMNOwatch, Somnomedics, Randersacker, Germany) to record physical activity during retention intervals. Visual inspections of actigraphy recordings indicated that participants of the sleep group slept while participants of the wake group did not fall asleep during the retention intervals.

### Statistical analyses

Memory performance was assessed using the standardized recognition accuracy d´ (d prime, i.e. standardized hit rate minus standardized false alarm rate; [37]). An analysis of variance (ANOVA) was performed using a 2x2 design with the factor SLEEP (sleep vs. wake) and AGE (children vs. adults). Rating data of encoding sessions were analyzed by 2x2 ANOVAs with the factors SLEEP and AGE. Rating data obtained during retrieval sessions were analyzed using 2x2x2 ANOVAs including the factors SLEEP, AGE and the repeated measure factor TARGET (targets vs. distractors). Current mental state ratings were analyzed by 2x2x2 ANOVA including the factors SLEEP, AGE and the repeated measure factor SESSION (encoding vs. retrieval). Comparisons of single means were performed by t-tests for dependent or independent samples. Correlations analyses were performed using Pearson´s correlation coefficient.

## Results

### Memory performance

**Fig 1:**
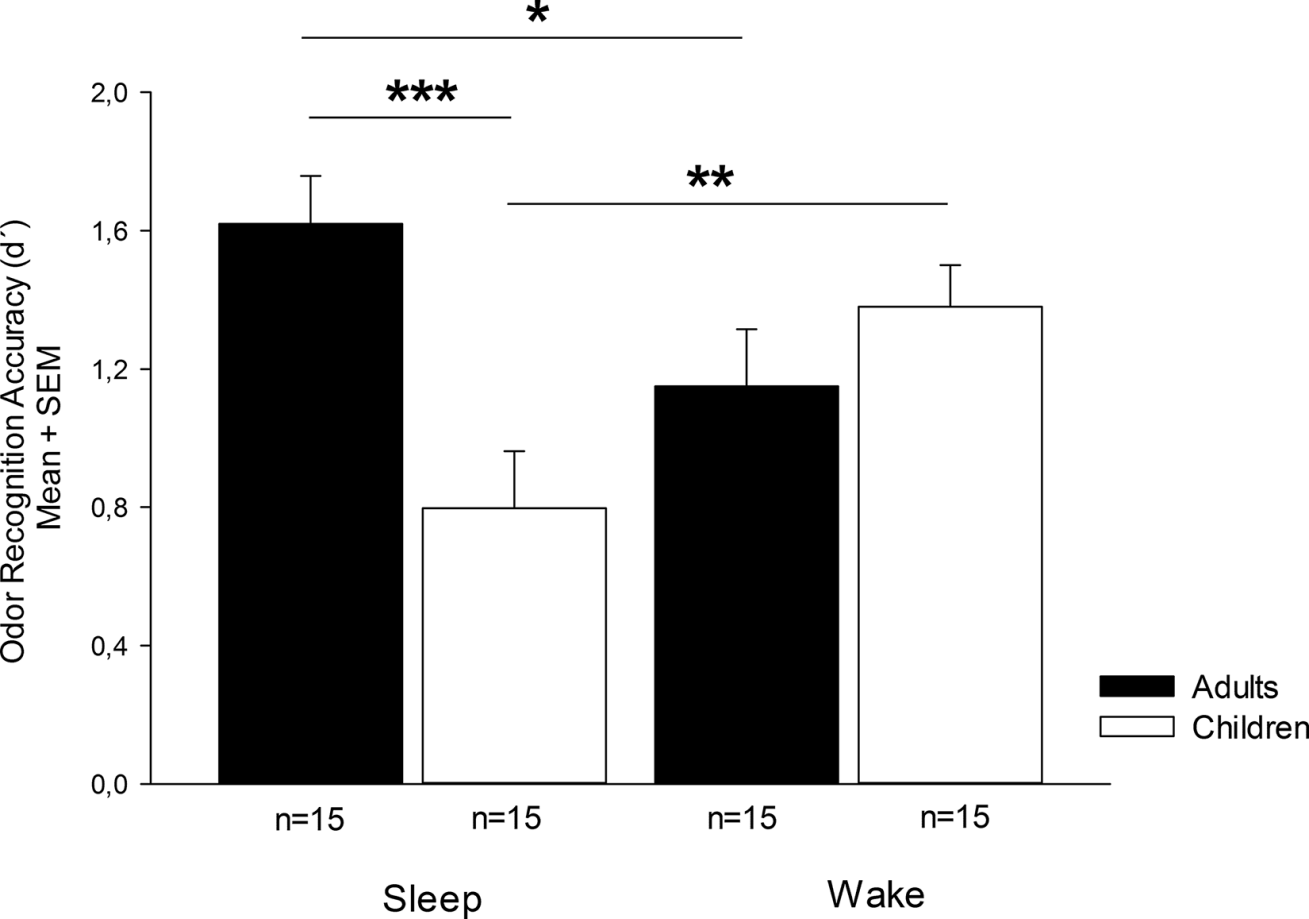
Odor recognition accuracy (d´) of the adult (black) and children (white) sleep and wake group; M, mean; SEM, standard error of means, *, p < .05; **, p < .01; ***, p = .001.

The analysis of memory performance revealed no main effect of the consolidation condition SLEEP [F(1,56) = 0.1, p = .7] and a marginally significant main effect of AGE [F(1,56) = 3.9, p = .052], indicating that adults generally displayed a higher recognition performance than children (see also Table 1 and Fig 1). The interaction SLEEP x GROUP, however, was significant [F(1,56) = 12.5, p = .001]. Subsequent t-tests for independent samples revealed that the adult sleep group performed better than the adult wake group [sleep: 1.6±0.14; wake: 1.1±0.15; sleep vs. wake: t(28) = 2.2, p = .038]. In contrast, recognition performance in the children wake group was higher than in the children sleep group [sleep: 0.8±0.16; wake: 1.4±0.12; sleep vs. wake: t(28) = 2.8, p = .008]. While recognition performances in the wake condition did not differ between children and adults [t(28) = 1.1, p = .270], group differences in the sleep condition became significant [t(28) = 3.8, p = .001].

### Odor rating

Encoding: Main effects for AGE revealed that children rated odors as being less intense [children: M = 5.1, SD = 1.0, adults: 6.2, SD = 0.8, F(1,56) = 24.2, p < .001] and less familiar [children: M = 4.4, SD = 1.3, adults: 5.9, SD = 0.6, F(1,56) = 1583.0, p < .001, see also Table 2] than adults. No further main effects (p < .4) or interactions (p < .176) reached significance; the same was true for positive or negative valence (p>.3).

**Table 2:** Odor rating data.

			Adult		Children	
			Sleep Group	Wake Group	Sleep Group	Wake Group
			M (SEM)	M (SEM)	M (SEM)	M (SEM)
Learning	Targets	Intensity	6.23 (0.21)	6.25 (0.22)	4.88 (0.23)	5.27 (0.28)
		Pleasantness	4.86 (0.18)	5.02 (0.14)	4.64 (0.34)	4.79 (0.28)
		Unpleasantness	2.30 (0.24)	2.31 (0.32)	2.15 (0.31)	1.91 (0.29)
		Familiarity	6.03 (0.17)	5.73 (0.15)	4.17 (0.32)	4.58 (0.34)
Retrieval	Targets	Intensity	6.38 (0.21)	6.23 (0.21)	4.98 (0.26)	5.49 (0.3)
		Pleasantness	4.96 (0.2)	4.80 (0.17)	4.65 (0.37)	5.01 (0.25)
		Unpleasantness	2.20 (0.24)	2.19 (0.26)	2.51 (0.34)	2.17 (0.28)
		Familiarity	6.18 (0.26)	5.55 (0.27)	4.43 (0.47)	5.12 (0.35)
		Confidence	5.95 (0.19)	5.38 (0.29)	5.23 (0.36)	5.33 (0.51)
	Distractors	Intensity	6.09 (0.23)	6.07 (0.23)	5.03 (0.26)	5.37 (0.3)
		Pleasantness	4.81 (0.18)	4.71 (0.24)	4.45 (0.42)	4.57 (0.32)
		Unpleasantness	2.35 (0.28)	2.29 (0.3)	2.41 (0.31)	2.50 (0.31)
		Familiarity	5.61 (0.19)	5.22 (0.3)	4.03 (0.39)	4.15 (0.45)
		Confidence	5.54 (0.32)	5.21 (0.22)	4.84 (0.39)	5.43 (0.41)

Note: ratings range from 0 = “not at all” to 8 = “maximum”

Retrieval: As shown by the main effect of AGE, children rated odors (targets and distractors) again as being less intense [children: M = 5.2, SEM = .19; adults: M = 6.2, SEM = .15; F(1,56) = 15.7, p < .001] and less familiar [children: M = 4.4, SEM = .28; adults: M = 5.6, SEM = .18; F(1,56) = 13.3, p = .001] than adults. Main effects of TARGET revealed that target odors were rated as being more intense [target: M = 5.8, SEM = .14; distractors: M = 5.6, SEM = .14; F(1,56) = 4.7, p = .035], more pleasant [target: M = 4.9, SEM = .13; distractors: M = 4.6, SEM = .15; F(1,56) = 8.1, p = .006], and more familiar than distractor odors [target: M = 5.3, SEM = .19; distractors: M = 4.8, SEM = .19; F(1,56) = 32.3, p < .001]. In addition, the interaction TARGET x SLEEP x AGE with respect to familiarity rating reached significance [F(1,56) = 4.0, p = .050]. Subsequent t-tests revealed that the sleep group, but not the wake group, rated target odors as being more familiar than distractors in adults only [sleep: t(14) = 4.0, p = .001; wake: t(14) = 1.7, p = .120]; in children’s groups, ratings were vice versa: only children in the wake group but not in the sleep group rated target odors as being more familiar [sleep: t(14) = 1.7, p = .114; wake: t(14) = 4.7, p < .001]. There were no further significant main effects or interactions with regard to intensity, arousal, pleasantness, unpleasantness, familiarity, or confidence ratings (p>.05).

### Sleep data

According to self-ratings, all participants in the sleep condition slept during the night (mean duration of sleep: M = 8.2h, SEM = 0.21), and no participant of the wake condition took a nap during daytime (see Table 1). Self-estimations of sleep duration revealed that children of the sleep group slept longer than adults of the sleep group [adults: M = 7.5h, SEM = 0.25, range: 5.5–9.25h; children: M = 8.9, SEM = 0.23, range: 7.5-10h; t(28) = 4.0, p < .001]. This sleep behavior was confirmed by actigraphy measurement [total sleep time: adults: M = 7.8h, SEM = 0.27, range: 5.5–9.5h; children: M = 8.8h, SEM = 0.27, range: 7.5–10.3h; t(28) = 2.5, p = .019].

### Correlation analyses

**Fig 2:**
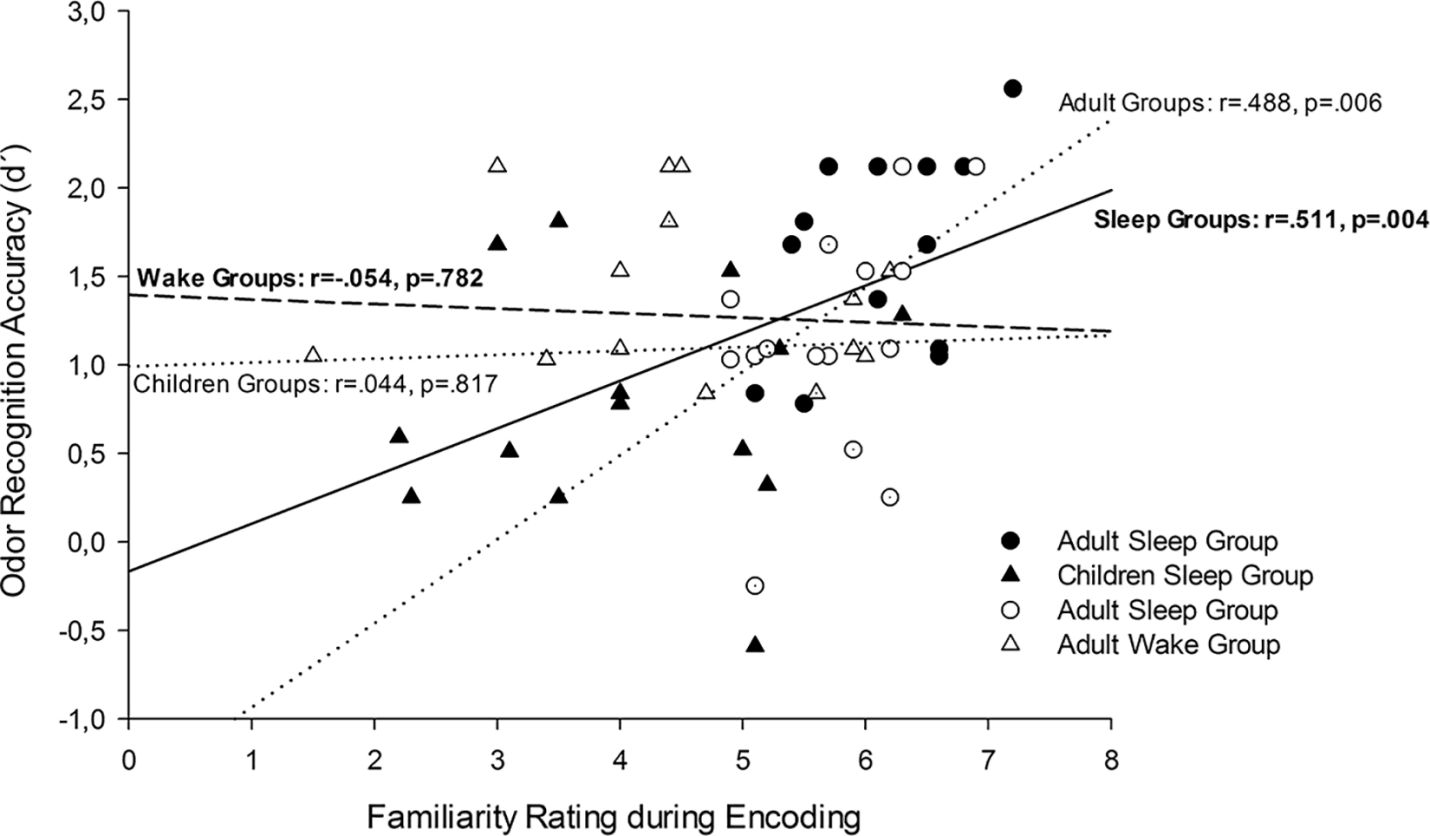
Correlations between familiarity ratings during encoding session and odor recognition accuracy during retrieval session; black symbols and solid regression line refer to the sleep groups [containing children (triangles) and adult (dots) data]; white symbols and the dashed line refer to the wake groups [(also containing children (triangles) and adult (dots) data]. Dotted lines refer to regression lines for the merged adult and for the merged children groups. Please note that correlation coefficients only differ significantly between the sleep and the wake groups (p = .023) but not between the children and the adult groups (p = .072).

Correlation analyses revealed that memory performance was associated with familiarity ratings during the encoding session (r = .285, p = .027, see also Fig 2). A group aggregation with respect to SLEEP showed that memory performance was correlated with familiarity ratings only in the sleep group (r = .511, p = .004) but not in the wake group (r = -.053, p = .782); correlation coefficients of sleep and wake group differ significantly (z = 2.27, p = .023). A separation according to AGE revealed that memory performance was correlated with familiarity ratings during encoding in adults (r = .488, p = .006) but not in children (r = .044, p = .817); here, the correlation coefficients only tended to be significantly different (z = 1.8. p = .072). Correlation coefficients did not reach significance in any of the four single groups (p < .086).

### Manipulation check

When participants were asked if they suspected being part of a memory study, five children of the sleep group and another six children of the wake group answered “yes”. Confidence ratings (from 0–100) of these children were M = 33 (range: 10–60) in the sleep group and M = 38 (range: 10–50) in the wake group. Four adults of the sleep group (confidence rating: M = 92, range: 80–100) and two adults of the wake group (confidence rating: M = 75, no range) suspected being part of a memory study. In order to control for possible effects of being aware of a later odor recall, we excluded all participants from the analyses who thought they were part of a memory study. Regarding odor recognition performance, the interaction SLEEP x GROUP, however, still was significant [F(1,39) = 11.5, p = .002]. Post-hoc t-tests revealed that the remaining adults of the sleep group again performed significantly better than children of the sleep group [t(19) = 3.6, p = .002] while the remaining children and adults in the wake groups still did not differ [t(20) = 9.4, p = .411). Likewise, adults of the sleep group still performed significantly better than adults of the wake group [t(22) = 2.7), p = .013] while children of the sleep group performed worse than children of the wake group [t(17) = 2.1, p = .048]. Besides the interaction, the main effect for SLEEP remained insignificant [F(1,39) = 0.02, p = .9], but the main effect for GROUP was significant now [F(1,39) = 5.5, p = .024]. With respect to familiarity ratings during retrieval, the interaction TARGET x SLEEP x AGE lost statistical power but was still marginally significant [F(1,39) = 3.9, p = .054]. Post-hoc tests showed again that only adults of the sleep group rated target odors as being more familiar than distractor odors [t(10) = 4.4, p = .001] but not adults in the wake group [t(12) = 1.3, p = .229]. Again, the opposite result was found in the remaining children [sleep: t(9) = 1.0, p = .331; wake: t(8) = 3.6, p = .007]. Correlation between familiarity ratings during encoding and d´ was still significant in the sleep group (r = .519, p = .016) and but remained insignificant in the wake group (r = -.1, p = .659).

### Control variables

To control for possible circadian effects of mood, tiredness, and arousal on memory performance, rating data assessed during encoding and retrieval sessions were compared between groups (see also Table 3). With regards to mood ratings, there was no main effect for SESSION (p = .158), AGE (p = 1), or SLEEP (p = .180). While the interactions SESSION x AGE [F(1,56) = .3, p = .569] and SESSION x SLEEP [F(1,56) = .1, p = .776] were not significant, the interaction SESSION x AGE x GROUP showed a trend towards significance [F(1,56) = 2.9, p = .092]. A decomposition of this 3-way interaction according to GROUPS did not reveal any significant main effects or interactions in the sleep groups (p>.165) or in the wake groups (p>.162).

**Table 3:** Rating data of current mental state.

		Adults		Children	
		Sleep Group	Wake Group	Sleep Group	Wake Group
		M (SEM)	M (SEM)	M (SEM)	M (SEM)
Learning	Mood	4.3 (021)	4.3 (0.16)	4.0 (0.24)	4.5 (0.17)
	Tiredness	3.3 (0.25)	4.3 (0.21)	3.1 (0.25)	3.2 (0.39)
	Arousal	3.9 (0.21)	4.2 (0.14)	3.7 (0.31)	3.5 (0.22)
Retrieval	Mood	3.9 (0.25)	4.3 (0.15)	4.1 (0.24)	4.2 (0.22)
	Tiredness	3.2 (0.17)	3.1 (0.26)	2.8 (0.24)	3.5 (0.32)
	Arousal	3.6 (0.21)	4.0 (0.17)	3.5 (0.34)	3.7 (0.23)

Note: ratings range from 1 = “lowest degree” to 6 = “highest degree”

The analysis of current tiredness ratings revealed a marginal main effect for SESSION [F(1,56) = 3.7, p = .058), a significant main effect for AGE [F(1,56) = 4.4, p = .026), and a marginally significant main effect for SLEEP [F(1,56) = 3.2, p = .083]. In addition, there was a marginally significant interaction SESSION x AGE [F(1,56) = 3.7, p = .058], no significant interaction SESSION x SLEEP [F(1,56) = 0.6, p = .443], but a significant interaction SESSION x GROUP x AGE [F(1,56) = 5.4, p = .024]. A decomposition of this 3-way interaction according to SLEEP revealed that children and adults of the sleep group did not differ in tiredness ratings [interaction SESSION x AGE: F(1,28) = 0.2, p = .658); however, in the wake groups the interaction SESSION x AGE still was significant [F(1,28) = 5.6, p = .026). Post-hoc t-tests indicated that adults of the wake group rated themselves as being more refreshed during encoding than during retrieval [t(14) = 3.8, p = .002]; there was no difference in the tiredness ratings between learning and retrieval sessions within the children wake group [t(14) = 0.5, p = .628].

There were no significant main effects or interactions with respect to physiological arousal ratings (p>.113).

To statistically control for any influence of mood, tiredness and arousal on memory encoding or retrieval on accuracy performance, these variables were included as covariates in the ANOVA. In addition, the familiarity ratings during encoding were included as a further covariate to control for possible random effects of odor familiarity. Analyzing memory performance by using an ANCOVA, however, still revealed a significant interaction GROUP x AGE [F(1,49) = 10.3, p = .002], indicating that the current state or familiarity ratings did not affect the observed memory performances. All covariates failed to reach significance (p>.074). In particular, the covariates for tiredness during encoding (p = .679) and during retrieval (p = .745), as well as the familiarity ratings during encoding (p = .479) were not significant.

## Discussion

While the adult sleep group showed better odor recognition performance than the adult wake group, the opposite was true in children. Correlation analyses showed that the odor memory performance in the sleep group was predicted by familiarity ratings during encoding.

The memory data of the adult groups are in line with a huge body of literature which has revealed that sleep supports the consolidation of various memory systems [7,38,39]. Although olfaction is structurally and functionally closely related to memory processes [40,41,42], the role of sleep in odor memory remains elusive [43]. As has been shown in rats, SWS strengthens the associations between odors and aversive stimuli in classical condition paradigms [11,12]. Recent studies in humans highlighted that odors can interact with the sleep-dependent consolidation of declarative memory [13,14,44,45,46]. In these studies, object locations were learned while a certain odor was presented. Applying the same odor during post-learning sleep improved the sleep-dependent memory consolidation, and object locations were better memorized the following morning. So far, only one human study investigated the role of sleep in remembering odors themselves [15]: Adults were explicitly instructed to learn odors in order to be able to recognize them among others in later retrieval sessions in a repeated measure design. Odors were recognized better after 3-hours of SWS-rich sleep than after wakefulness. However, in intentional odor learning paradigms, as used by Gais and colleagues [15], the olfactory stimuli may also act as contextual cues for verbal labels or mental images stored during encoding [17,47,48]. By introducing an incidental odor learning paradigm with unannounced subsequent recognition, we were able to focus on the recognition of olfactory sensation itself. As adult data have shown, sleep can support the recognition of odors, even in the absence of explicit memory instructions. Adults´ memory data were confirmed by familiarity ratings during the retrieval session: target odors were rated as being more familiar than distractors, a result which was even more pronounced in the sleep than in the wake group. Please note that adult groups did not differ in familiarity ratings during encoding. [36]

In contrast to adults, sleep in children did not improve the consolidation of odor memory. As the analysis of control variables showed, these results cannot be ascribed to circadian effects of mood, tiredness, or arousal. Regarding the comparable memory performance in the children and adult wake group, our data indicate that sleep even attenuated odor memory performance in children. In accordance with this, only children in the wake group but not in the sleep group rated target odors as being more familiar than distractor odors. At first glance, these results seem to be counterintuitive. As mentioned above, the SWS is assumed to be the critical sleep stage supporting odor memory [43]. In the case of declarative memory, children benefit from SWS just as adults do [23,27,49]. Based on the fact that children display more SWS than adults [20,21], one could expect that children perform at least on an equal level of odor recognition after sleep. A similar negative effect of sleep on consolidation was observed in the domain of motor memory in children. Children showed worse motor memory performance after a sleep interval than after wakefulness [26,27,28]. In that case it was argued that post-learning sleep did not foster the consolidation in children due to a lower level of pre-experiences [6]. The integration of sensory inputs into existing memory systems during sleep depends on the presence of pre-existent representations [30]. Several studies have shown that schema-conform information is consolidated better during sleep than information that not does fit into a pre-existing schema [29]. Here, theta oscillations (5-8Hz) during the rapid-eye-movement (REM) sleep predicted the benefit of sleep on the consolidation of schema-conform memory [50]. Familiarity rating data clearly indicate that children were less experienced with the olfactory stimulus material [51,52]. Correlation analyses confirmed that the more familiar odors were, the better they were recognized. This association was significantly more pronounced in the sleep condition than in the wake condition (data separation according to age revealed only marginally significant differences in correlation coefficients between adults and children). Therefore, pre-knowledge appears to be less relevant for odor memory consolidation during wakefulness but seems to be the critical predictor of whether sleep fosters or attenuates odor memory. By including familiarity ratings of encoded odors as covariates, we ensured that differences in recognition performance within the children groups and within the adult groups were not due to random effects of group differences in odor familiarity. Hence, our data indicate that sleep might not only be characterized by a unidirectional function of memory stabilization but may also destabilize memory engrams for unfamiliar (and rather irrelevant) stimulus material. However, it remains to be confirmed whether becoming familiar with odors reverses the sleep-associated loss in odor memory as observed in children. At the same time, using unfamiliar odors in adults should prove that less familiarity with odors reverses the sleep-associated gain in memory, independently of age.

Indeed, by the age of eight years, not all olfactory functions are refined [53,54], and the olfactory knowledge (odor naming, stability of odor naming, odor identification) as well as odor memory improves with maturation [55,56,57,58]. In addition to an age-dependent intensity and familiarity rating, adults rated odors as being more pleasant which also might influence later memory performance [59]. However, since memory performance in the adult and children wake groups did not differ, it seems unlikely that different memory performances of the sleep groups can be ascribed to maturational changes in odor memory. As confirmed by actigraphy, none of the participants of the wake group took a nap, and sleep durations of sleep groups were typical for the age groups [60,61].

As has been seen in adults, the expectation of whether or not stimulus material is relevant for later memory retrieval is a critical precondition for enhanced memory consolidation during post-learning sleep [62]; only participants who were aware of subsequent retrieval showed better memory performance after sleep [63]. In our study, all participants were naïve to the aim of the study. Some participants reported that they suspected being part of a memory study; however, the exclusion of these participants from the analyses did not change the memory results. Therefore, our data cannot be attributed solely to effects of explicit encoding strategies such as verbal labelling or mental imaging.

Half of our participants performed the encoding in the morning (children at 8 a.m., adults at 9 a.m.) and the other half in the evening (children at 8 p.m., adults at 9 p.m.). In all groups, the retrieval was performed after a 12h retention interval either in the morning or in the evening. This might lead to the concern that memory performance could have been affected by different circadian effects. A) Odor performance in the morning in general is different from odor performance in the evening. Indeed, there is one study which has reported that some electroencephalographical responses to chemosensory stimuli show circadian patterns [64]. Behavioral responses to olfactory sensations (odor discrimination), however, were not influenced by daytime [65]. If in any case a daytime-dependent change in odor performance occurred in our data, then this would be true for all groups: if e.g. odor performance were affected in the morning, then participants of the wake group would be disadvantaged during encoding, but participants of the sleep group would show similar disadvantages during retrieval in the morning time. Please also note that the olfactory sensitivity was proved in all participants prior to each session. B) Adults and children were tested at different time points of their sleep-wake cycle. The most significant changes in circadian rhythms can be found during puberty where the sleep-wake cycle becomes more and more delayed [18]. In that way too, the optimal time of cognitive performance shifts towards later in the day [66]. By the age of 20, however, these changes reverse, and the sleep-wake cycle shifts to earlier times again [19]. To address for age-dependent changes in the sleep-wake cycle (usual time to wake up in adults was on average about 70 minutes later than in children), we invited adults one hour later than children to the experimental sessions. By including self-ratings regarding the current mental state during the encoding and retrieval sessions, we statistically controlled for possible daytime-dependent effects of mood, tiredness, or arousal. Please note again that children and adults in the wake group showed comparable memory performance, and that differences between encoding and retrieval in tiredness ratings were only significant within the adult wake group. After including these variables as covariates, the memory results did not change. Therefore, we doubt that circadian effects affected our data considerably. However, only additional control groups for an immediately recall (see also [67]) could have controlled for circadian effects on memory performance optimally. Another limitation is that no sleep EEG was recorded and we have no information about any physiological mechanisms behind the observed interaction between memory and age during sleep. Therefore, further polysomnographic studies are required to investigate whether SWS or REM sleep supports memory for familiar odors and destabilizes memory for unfamiliar odors. In particular, it needs to be confirmed whether becoming familiar with odors reverses the sleep-associated loss in odor memory as observed in children. In the same way, further functional imaging studies are required to elucidate the possible underlying neural processes of developmental changes in odor memory consolidation during sleep. Also, possible gender-effects of olfaction [68,69] should be taken into account by including female participants.

Taken together, we found that sleep supports the recognition of incidentally encoded odors only in adults; in children, who were less familiar with odors, sleep had the opposite effect. Pre-experience might be a crucial condition for sleep-dependent memory stabilization/destabilization.
